# A citation study of earth science projects in citizen science

**DOI:** 10.1371/journal.pone.0235265

**Published:** 2020-07-16

**Authors:** Sten F. Odenwald

**Affiliations:** 1 ADNET Systems Inc., Bethesda, Maryland, United States of America; 2 NASA Space Science Education Consortium, Goddard Space Flight Center, Greenbelt, Maryland, United States of America; Universidad de las Palmas de Gran Canaria, SPAIN

## Abstract

A citation study of a sample of earth science projects in citizen science from the FedCats Catalog was undertaken to assess whether citizen science projects are as productive and as impactful as conventional research that does not employ volunteer participation as a part of their data gathering and analysis protocols. From the 783 peer-reviewed papers produced by 48 projects identified from project bibliographies, 12,380 citations were identified using the Web of Science archive and their citation search engine to the end of 2018. Various conventional productivity and impact measures were applied including the Impact Factor, H and M-indices, and entry into the Top-1000 papers in cited research. The earth science projects tend to under-perform in terms of Impact Factor (IF = 14–20) and the M-index (M<0.5) but perform at the level of a ‘tenured professor’ with <H> = 23. When compared to non-citizen science research in general, there is a ten-fold higher probability of the earth science papers reaching the Top-1000 threshold of most-cited papers in natural science research. Some of the reasons for the lower performance by some indicators may have to do with the down-turn in published papers after 2010 for the majority of the earth science projects, which itself could be related to the fact that 52% of these projects only became operational after 2010 compared to the more successful ‘Top-3’ projects, whose impacts resemble the general population of non-citizen science research.

## 1.0 Introduction

Among the many concerns that scientists raise to the citizen science (hereafter CS) approach for conducting scientific research are whether the results will actually be meaningful to the scientific community at large. This consideration relates to questions about whether novice, non-professional participants are capable of delivering high-quality data to the Principle Investigator or project team [1,2]. Labor and software development costs also have to be controlled, which are associated with setting up and operating a CS project [3]. The bottom-line concern for most scientists, however, is whether the effort will ultimately lead to publishable results that will advance scientific knowledge in some measurable way.

Contemporary bibliometric studies use a variety of indices to gauge productivity and impact including counts of publications and citations [4]. This is a straight-forward exercise in using an individual’s research bibliography and simply counting the number of papers that cite items in the bibliography. This approach was used, for example, by [5] who investigated the impact of space science CS projects (hereafter SSCS) to find that they can, indeed, make significant contributions to scientific research in peer-reviewed journals.

This paper is a follow-on to the previous investigation in space science, but this time within the project areas of earth science. The objective as in the space science study is to use traditional citation analysis methodology to investigate the impact of earth science CS (ESCS) projects compared to non-ESCS projects.

## 2.0 Quantifying productivity and impact

One of the easiest indices to create is simply the cumulative number of published papers, P, over a particular span of years. This ‘productivity’ index has been used since at least the 1960’s and its historical applications to academic research has been discussed by [5]. However, a simple count of papers, P, as a measure of productivity is generally considered inadequate as discussed by [6,7]. The specific issue is that this counting does not distinguish by the quality or impact of the papers, and it penalizes younger researchers for not having accumulated as many papers as older investigators. An obvious normalization to P is to divide the paper counts by the number of years the publication has been circulating leading to the secondary measure of publications/year (PPY). This corrects for older researchers or projects accumulating more publications than younger ones.

A second measure of productive output is the number of times a paper is cited by others in the community described historically by [8]. A citation index (CI) is simply a count of the number of documents that cite a target document from the time of its publication to the current year. The use of the CI in academic journals to assess their merit or impact is a relatively recent application [9] often used in assessing a candidate’s suitability for tenure, or identifying influential, core documents within a specific research genre. CIs as simply a straight count of the number of citations, can be strongly influenced by authors who self-cite their own research work, which artificially increases the apparent impact of an author’s research. Although it may seem as though self-citations are a negative factor, in fact this practice is a normal part of scientific research that allows the current research to be seen in the context of the researcher’s previous intellectual work and evolution. Nonetheless, bibliographic studies generally see self-citations as a negative factor to be mitigated [10]. The rate is highest exceeding 30% for papers with small numbers of citations, but approaches 15% or less for citations per paper of > 50 [5,11,12]. In this study of ESCS projects, I make no correction for this effect due to the large numbers of papers involved, but expect that the rates will be commensurate with non-ESCS papers and therefore not significantly affect any comparison between ESCS and non-ESCS results.

Although a bibliography of published works can be current up to the present year, citations of these papers may not appear until several years after publication. The consequence of this lag is that the citations to recent publications in a bibliography will be under-counted relative to older papers. Awareness of this effect will be taken into consideration when the various metrics are evaluated and interpreted. In general, the citation trends of projects will be carefully scrutinized for papers published since 2015 where this effect will be the most prominent.

A more worrisome issue is that the citation database itself may be incomplete. A study by [13] found that the Web of Science [14] (hereafter WoS), Scopus and Google Scholar citation archives reported significantly different citation rates, leading to different assessments of research impact, and also significant variations between disciplines. The discrepancies were the highest for social science disciplines and the lowest for scientific disciplines, with WoS being the most popular citation resource.

In addition to the citation index CI, one straight-forward secondary index is the citation per paper ratio also called the Impact Factor, IF, [15,16,17,18] defined by dividing the cumulative number of citations C for an ensemble of papers up to a given year by the cumulative number of peer-reviewed papers, P [18] such that IF = C/P. As with other counts of citations, one must apply the principle of *caveat emptor* (e.g. Let the buyer beware). Although the number of publications can be considered to be complete up to the current year, the number of citations can be seriously incomplete for younger papers compared to papers that have been in circulation for many years. As the citation counts approach the current year, their numbers will invariably start to decline as recent publications have not had enough time to circulate and be cited.

An additional higher-order measure of impact is the H-index originally suggested by [19] and later by [20] as a means for determining the impact of published research by theoretical physicists within their community. The H-index was primarily intended to rank individual investigator’s impact, with H = 12 being a suggested threshold for advancement to tenure in the ‘hard sciences’ [19]. The achievement of H-values in the double-digits or higher is considered a successful and impactful scientific accomplishment. Although this index was originally used to assess individual researcher’s performance, it has also been used in various other settings. For example, [21] use the H-index among other citation indices to rank universities and colleges in terms of the quality of their research. Entire countries have been ranked according to their equivalent H-index [22], as has internet media outlets such as YouTube [23].

The H-index is defined for an author as having published ‘H’ papers that have each been cited at least ‘H’ times. It is computed by taking an author’s rank-ordered citation count among the author’s publication list and finding the H-th rank such that the rank matches the number of citations. For example, if a project, institution or author has 5 publications with citations of 34, 12, 8, 4, and 2, the H-index will be 4 because the 4^th^ ranked publications has 4 citations. A number of criticisms of the H-index have been offered such as those reviewed by [24,25]. For example, it does not discriminate among the citation practices between fields [26], and it can be artificially manipulated via self-citations [27]. Also, the H-index makes no corrections or normalizations for the age of the researcher/project or the number of co-authors in the papers, which would be significant issues in comparing one project with another. The H-index of an author increases in time as they publish more papers. The age-effect can, for example, be removed by dividing the H-index by the number of publishing years to define what some bibliometricians call the M-index [19,28]. The co-author effect, meanwhile, can be normalized-out by first dividing a paper’s citations by the number of authors, and then computing the H-index from the resulting rank ordered values according to [13,29].

As a means for testing the hypothesis that ESCS projects have similar impact and productivity to non-CS projects, I will use the indices P, CI, CPY, IF and the H and M-indices to compare the ESCS, and non-ESCS research to determine whether CS research is statistically different from conventional research in earth science and in what specific ways. I will also compare ESCS projects with SSCS projects to see if earth science and space science CS projects have statistically similar or different impacts given that they both involve the CS approach in analyzing data.

## 3.0 Methods

### 3.1 Preparing the CS sample

Generally, citation studies identify a group of papers within a uniform subject matter area and then proceed to use various tools such as the WoS to tabulate the citations to these papers. In a previous study of SSCS projects by [5] this step was greatly facilitated by the relatively small number of CS projects that fit into the category of having subject matter outside Earth’s atmosphere and designated as either space science or astronomy-related topics. For this study, the identification of papers in the ‘Earth Science’ category represented a vast array of potential subject matter possibilities including biology, geology, ornithology and meteorology among other disciplines.

To narrow the list to a manageable number, I used the Federal Crowdsourcing and Citizen Science Catalog [30] (hereafter FedCat), which provides a government-wide catalog of 425 citizen science projects as of December 1, 2018. These projects are nominally supported by scientists who work at the various agencies such as NASA, USGS, US Forestry Service, etc. There were 132 (31%) identified as operating ESCS projects, along with 18 (4%) SSCS projects. Bad or inoperative project URLs were encountered for nine (2%) projects. Catalog entries that were primarily announcements for software, data bases or apps were identified in 60 (14%) cases. In 34 (8%) cases, the projects were primarily educational, consisting of announcements for summer camps or classroom activities. There were 47 (11%) instances of the projects being primarily scientific studies with limited public participation (e.g. the number of volunteers that registered with a CS project to participate). Finally, 123 projects (29%) were identified as ‘BioBlitzes’, which are annual two-day, public participation events hosted by the National Park Service at parks across America to study biodiversity (birds, insects, etc). An additional three projects were considered unclassifiable and not included in this tally.

For the purposes of this study, only the projects in the earth science category were considered. Other CS cataloging services such as SciStarter also include non-governmental projects and would be considered a complete, current list of these projects of which the FedCat listing is a subset. For example, the National Audubon Society sponsors the *Hummingbirds at Home* project, listed in SciStarter [31] but not in FedCat. Consequently, this citation study should be properly considered to be a complete study of government-supported or government-affiliated earth science, CS projects.

### 3.2 Project bibliography and citation statistics

Having identified the projects in the ESCS sample, a fairly lengthy and iterative process of identifying the publications from each project ensued. In some instances, this publication search was greatly facilitated by projects that provided a bibliography at their website. For many other projects, Google was used extensively to identify publications by using keywords such as ‘citizen science’ followed by the project name or the name of the project PI. This assumed, quite reasonably, that publications by a project would include mentions of the project name and/or the name of the creator/PI of the project. The FedCat and SciStarter catalogs in the majority of the cases provided names of the PIs, although some projects chose not to do so. The result of this online publication search yielded 94 earth science projects from the ESCS sample that had identifiable publications and also participation level estimates. There were an additional 18 ESCS projects that had publication lists but no official estimates of participation levels. This combination of 108 programs are considered ‘Active’ and formed the basis for the analysis to follow.

There were also 20 ‘silent’ earth science projects that could not be identified with publications beyond the project website page and institutional announcement. Contacts were attempted with the project PIs to inquire about project publications and participation rates. The result was that no further information was available for these projects, which may indicate that they were inactive or discontinued.

The 108 active ESCS projects, representing the efforts of some 4 million participants, reported 5001 publications including scientific research papers, conference abstracts, popular articles in newspapers and magazines, books, and dissertations. Virtually all of the ‘Education’ publications were from *The GLOBE* program. The current study focused only on articles published in peer-reviewed science journals since the goal of this study is to assess the scientific impact of citizen science research in ESCS projects. Conference abstracts were often duplicative of the identified peer-reviewed research papers, so this study focusses on the 783 papers that constitute the ‘Research’ category. These papers were published in 352 journals of which the Top-36 journals that published more than 4 papers accounted for 45% of the published papers. Not included in this research publication list are the additional 198 research documents that could be described as reports, which included project summaries and evaluations (e.g. ‘*GLOBE 10-year Evaluation; Prepared by SRI International for The GLOBE Program*’) and other similar documents and books.

Table 1 summarizes the available publications, participant sizes and current age of the project by the end of 2018. Column 2 indicates the federal agency identified as the project’s sponsor. Column 4 is the inception year of the project followed by column 5 in which the age of the project to 2018 is calculated with the inception year counted as Year 1. Column 6 gives the log_10_ of the number of participants. Column 7 is the total number documents found for the project including research publications (column 8), reports (column 9), popular essays (P: column 10), conference abstracts (C: column 11), books (B: column 12), educational reports (E: column 13), and dissertations. Across the 108 active projects, the median age of these active-CS projects (column 5) is 6 years, and the median number of participants is about 1,000. About half of the projects (48) report a combined 783 articles (column 8) in peer-reviewed journals for a median of about two papers published per project among these programs. The total number of participants among the active projects is about 4.3 million. There have also been a total of 110 books and 42 Masters/PhD dissertations generated by this cohort of projects. Note that the large number of documents for the *Smithsonian Digital Volunteers* project reflects the fact that each document transcribed by volunteers becomes part of the permanent Smithsonian archive and so represents a single ‘published’ document. The table is ordered by decreasing numbers of peer-reviewed publications (col. 8).

**Table 1:** Active ESCS programs.

(1)	(2)	(3)	(4)	(5)	(6)	(7)	(8)	(9)	(10)	(11)	(12)	(13)
	Agency	Project	Year	Age	Log(n)	Doc	Re	Rp	P	C	B	E
1	NPS	Christmas Bird Count	1900	118	4.9	346	306	25	3	3	9	
2	USFWS	eBird	2003	15	5.6	239	196	5	22	11	5	
3	NASA	The GLOBE Program	1994	24	5.1	572	61	15		89	81	326
4	NOAA	CoCoRaH	1998	20	4.3	29	26	2			1	
5	USDA	Snow Surveyors	2014	4	2.0	28	26			1		
6	NOAA	WormWatch	1999	19	3.0	26	19	1	6			
7	NOAA	COSS	2005	13	3.0	23	15	4	1		3	
8	NPS	Woodcock Survey	1968	50	2.8	30	13	14		1	2	
9	NSF	Project BudBurst	2007	11	3.5	11	10	1				
10	USGS	NA Amphibians	1997	21	3.0	11	10				1	
11	USGS	Quake Catcher Network	2008	10	3.4	10	10					
12	NPS	Raptors	1983	35	3.1	16	8	3	3		1	1
13	USGS	Did You Feel It?	2003	15	6.3	12	8	1	1	1	1	
14	NASA	GLOBE at Night	2009	9	4.3	68	7	1	57	2		1
15	NPS	Common Loon Project	2005	13	3.3	13	6	2	5			
16	USFS	Backyard Bark Beetles	2013	5	2.8	7	6	0	1			
17	NOAA	Beach Watch	1993	25	2.0	24	5	2	17			
18	NSF	Foldit Game	2015	3	4.8	8	5	1	2			
19	EPA	Gardenroots	2008	10	2.0	3	3					
20	EPA	Lakes of Missouri	1992	26	2.0	2	2					
21	EPA	The Secchi Dip-In	1994	24	3.5	9	2	5	1	1		
22	NASA	Landslide Reporter	2018	0	1.6	2	2	0	0	0		
23	NASA	MAPPD	2016	2	2.7	4	2	0	1		1	
24	NIH	Barcode Long Island	2014	4	3.8	2	2	0				
25	NOAA	Cyclone Center	2012	6	3.6	5	1		4			
26	NOAA	Marine DMA	2012	6	2.7	8	2	3	1	1	1	
27	NOAA	SKYWARN	1975	43	5.6	12	2		6	2	1	
28	NSF	The Evolution Project	2016	2	3.1	3	2	0	1			
29	NSF	WiEye	2011	7	4.8	23	2	0		21		
30	USDA	CARM	2012	6	3.0	4	2	1	1			
31	USFWS	Alaska Bats	2011	7	2.0	8	2		5	1		
32	USGS	Insect Monitor	2012	6	2.4	22	2	0	20			
33	USGS	Michigan AMBLE	2011	7	1.6	9	2		6	1		
34	EPA	Georgia Adopt-a-Stream	2013	5	2.0	4	1	3				
35	EPA	LEOnet	2012	6	2.3	5	1	2	2			
36	NIH	EyeWire	2013	5	4.9	8	1	0	7			
37	NOAA	CWOP	2000	18	4.5	4	1		2	1		
38	NOAA	Hui o ka Wai Ola. . .	2016	2	1.4	3	1		2			
39	NOAA	ISeeChange	2012	6	3.7	10	1	0	9			
40	NOAA	Old Weather	2012	6	4.3	6	1	0	5			
41	NPS	Cascades Butterfly Project	2008	10	2.3	4	1	2	1			
42	NPS	Dragonfly Mercury Project	2015	3	3.6	10	1	8			1	
43	NSF	Notes from Nature	2013	5	3.8	5	1	0	4			
44	NSF	Season Spotter	2015	3	4.1	1	1					
45	USFS	Gros Ventre Project	2009	9	1.4	1	1					
46	USGS	CyanoTRACKER	2015	3	2.0	8	1			7		
47	USGS	Bald Cypress Net	2010	8	2.0	4	1		1	1	1	
48	USGS	SEANET	2006	12	1.7	5	1		2	2		
49	NOAA	Urban Watch	1998	20	3.0	2		2				
50	USGS	CrowdHydrology	2010	8	3.9	8		2	6			
51	NASA	S'COOL	1997	21	3.5	50		2	1	46		1
52	NOAA	Horseshoe Count	1990	28	2.0	26		26				
53	EPA	GACS	2016	2	1.0	4		1	2	1		
54	EPA	Urban Waters	2014	4	2.0	1			1			
55	EPA	Arizona Water Watch	2017	1	2.3	2			2			
56	EPA	Cyanomonitoring	2013	5	2.5	8			5	3		
57	EPA	Smoke Sense	2017	1	3.7	7			7			
58	EPA	Summer on the Marsh	1987	31	2.9	1		1				
59	EPA	AirKeepers	2016	2	2.4	9			9			
60	FCC	FCC Speed Test	2009	9	5.4	2		1	1			
61	NASA	Floating Forests	2014	4	3.6	5		1	3	1		
62	NASA	GLOBE Adopt a Pixel	2018	0	3.6	5		1	4			
63	NASA	Mosquito Habitat Mapper	2017	1	3.8	3			2	1		
64	NASA	GLOBE Observer—Clouds	2016	2	4.3	4			4			
65	NASA	Image Detective	2012	6	2.3	4			3	1		
66	NASA	Picture Post	2012	6	2.0	67			67			
67	NOAA	CARIB Tails	2014	4	2.0	6			6			
68	NOAA	CrowdMag	2014	4	3.5	5				5		
69	NOAA	Elkhorn VWQ	1988	30	2.0	3		3				
70	NOAA	First Flush	2000	18	2.0	16		16				
71	NOAA	Florida MAP	2015	3	2.5	1			1			
72	NOAA	NWS-Coop.	1890	128	4.0	0						
73	NOAA	Nature's Notebook	2007	11	3.8	5		5				
74	NOAA	PhytoNet	2001	17	4.0	3		1	1	1		
75	NOAA	San Gabriel Turtles	2012	6	1.7	6			6			
76	NOAA	Main Beaches	1999	19	2.1	3		1	2			
77	NOAA	Steller Watch	2017	1	4.0	7			7			
78	NOAA	Stellwagen Stewards	1995	23	3.8	1			1			
79	NOAA	Hudson River Eels	2008	10	2.9	8		1	6	1		
80	NPS	DEW Picture Post	2005	13	3.4	4		1		3		
81	NPS	Kenilworth PP	2016	2	1.7	1			1			
82	NPS	Wood Thrush PP	2015	3	1.1	1			1			
83	NSF	Arizona BatWatch	2016	2	3.6	4			2	2		
84	NSF	Goose-Veg. Interactions	2014	4	2.0	3			1	2		
85	NSF	Coastal SEES	2017	1	1.0	3		3				
86	NSF	Habitat Network	2012	6	5.5	7			7			
87	NSF	Jungle Rhythms	2016	2	3.9	2			2			
88	NSF	Map of Life	2011	7	4.0	2			2			
89	NSF	Project Sidewalk	2012	6	2.6	5			4	1		
90	Smith	Parasite Project	2003	15	2.0	4			4			
91	Smith	Digital Volunteers	2013	5	4.1	2950						
92	USFS	Citizen Foresters	2014	4	1.3	0						
93	USFS	Atlanta Pollinators	2011	7	2.5	7		1	5	1		
94	USFS	Kaibab Forest	2017	1	2.2	5			5			
95	USFS	Migratory Dragonflys	2012	6	3.8	3		1	2			
96	USFS	Rare Carnivore Monitoring	2015	3	1.5	1		1				
97	USFWS	Pelican Project	2016	2	2.5	5		3	2			
98	USFWS	Condor Watch	2014	4	3.5	7		1	6			
99	USFWS	Bay Shorebirds	1997	21	2.0	4		1	2	1		
100	USFWS	Monarchs	2017	1	2.4	1			1			
101	USFWS	Arizona Feeders	2007	11	2.0	3			3			
102	USGS	North Am BM	1995	23	4.7	2			2			
103	USGS	DC&B Crickets	2012	6	2.6	4			4			
104	USGS	Did You See It?	2012	6	3.0	1					1	
105	USGS	iCoast	2014	4	3.3	7		1	3	3		
106	USGS	NYC Cricket Crawl	2009	9	2.0	3		3				
107	USGS	Loosestrife Volunteers	2003	15	1.7	2			1	1		
108	USGS	USGS/Adopt a Pixel	2013	5	2.2	1		1				

Abbreviations: General Aviation Citizen Science (GACS); Local Environmental Observer Nertwork (LEOnet); GLOBE Cloud Observation Project (S’COOL); Mapping Application for Penguin Populations and Dynamics (MAPPD); Citizen Weather Observer Program (CWOP); Coastal Observation and Seabird Survey (COSS); Community Collaborative Rain and Hail Network (CoCoRaH); Elkhorn Slough Volunteer Water Quality Project (Elkhorn VWQ); Florida Microplastic Awareness Project (Florida-MAP); Great Lakes Worm Watch (WormWatch); Marine Debris Monitoring and Assessment (Marine DMA); National Weather Service Cooperative Observer Project (NWS-Coop); Phytoplankton Monitoring Network (PhytoNet); San Gabriel River Sea Turtle Project (San Gabrial Turtles); Southern Maine Volunteer Beach Profile (Main Beaches); Stellwagen Sanctuary Seabird Stewards (Stellwagen Stewards); American Woodcock Singing-ground Survey (Woodcock Survey); Digital Earth Watch Picture Post (DEW Picture Post); Golden Gate Raptor Observatory (Raptors); Kenilworth Aquatic Gardens Picture Post (Kenilworth PP); NPS Wood Thrush Picture Post (Wood Thrush PP); Asynchrony in the timing of Goose-Vegetation Interactions (Goose-Veg. Interactions); Coastal SEES Collaborative Research (Coastal SEES); Chesapeake Bay Parasite Project (Parasite Project); Collaborative Adaptive Grazing Management Experiment (CARM); Greater Atlanta Pollinator Partnership (Atlanta Pollinators); Kaibab National Forest Citizen Science (Kaibab Forest); Migratory Dragonfly Pond Watch Project (Migratory Dragonflies); Acoustic Bat monitoring in Alaska (Alaska Bats); California Brown Pelican Citizen Science Project (Pelican Project); Delaware Bay Shorebird Project (Bay Shorebirds); Monarch Butterfly Integrated Monitoring (Monarchs); Southern Arizona Bat-Hummingbird Feeder Monitoring (Arizona Feeders); Butterflies and Moths of North America (NorthAm BM); Citizen Science Insect Monitoring (Insect Monitor); DC/Baltimore Cricket Crawl (DC&B Crickets); iCoast- Did the Coast Change? (iCoast); Lake Michigan AMBLE (Michigan AMBLE); North American Amphibian Monitoring Project (NA Amphibians); North American Baldcypress Swamp Network (Bald Cypress Net); Purple Loosestrife Volunteers (Loosestrife Volunt.); Seabird Ecological Assessment Network (SEANET); Georgia Adopt-a-Stream (Georgia-Adopt); GLOBE Mosquito Habitat Mapper (GLOBE Mosquito); GLOBE Observer–Clouds (GLOBE Clouds); Cascades Butterfly Project (Butterfly Project); Dragonfly Mercury Project (Dragonfly Project); Smithsonian Digital Volunteers (Digital Volunteers); Rare Carnivore Monitoring (Rare Carnivores)

Although there were 108 earth science projects in the FedCat, only 48 were found to have peer-reviewed publications. The remaining 60 programs in Table 1 had publications in non-refereed publications such as project reports, conference abstracts, popular articles and other resources. The apparent fact that about half of the active-ESCS projects do not report peer-reviewed publications is similar to what was found in the citation study by [5] for SSCS projects, and for the scientific output of major observatory facilities by [32,33] suggesting that some projects based upon well-designed scientific objectives and data bases nevertheless do not publish any findings, or at least not at the level of peer-reviewed scholarship. There are two distinct groups in this sample. The first group, which I will call ‘Top-3’, consists of the projects: *Christmas Bird Count* (306), *eBird* (196), and *The GLOBE Project* (61) that collectively account for 72% of the 783 peer-reviewed research papers. The second group, which I will call the ‘Lower-45’, consists of publications contributed by the remaining 45 projects totaling 219 papers. This investigation will treat these two groups separately to avoid biasing the discussion of the more typical project performance data from the more successful projects.

The Silent-ESCS projects with no identifiable publications are listed in Table 2. Their median age is three years with a median population size that appears to be about 100 participants though participation information is incomplete.

**Table 2:** Silent-ESCS programs.

(1)	(2)	(3)	(4)	(5)	(6)
Index	Agency	Project	Year	Duration	Participants
**1**	EPA	Berkeley Lab Range Hood Roundup	2014	4	
**2**	EPA	Chesapeake Monitoring Cooperative	2015	3	
**3**	EPA	Indigenous Observation Network	2006	12	300
**4**	EPA	IDAH2O Master Water Stewards	2010	8	150
**5**	NOAA	Team Ocean Science Diver Program	2015		
**6**	NPS	Rocky Mountain Christmas Bird Count	1900	118	
**7**	NPS	A.T. Seasons Phenology Project	2013	5	100
**8**	NPS	Citizen Science Track Trail	2017	1	50
**9**	NPS	Joshua Tree National Park Wildflower Watch	2015	3	767
**10**	NPS	Rocky Mountain Butterfly Project	1995	23	
**11**	NSF	Disease Ecology in birds	2016	2	
**12**	NSF	Lost Ladybug Project	2008	10	
**13**	NSF	Sentinels of the sounds	2016	2	70
**14**	NSF	WeDigFLPlants	2016	2	
**15**	Smith.	NC Candid Critters	2016	2	
**16**	USDA	Promoting Native Bee Health	2015	3	24
**17**	USDA	Broodmapper: Honey Bee Development	2012	6	
**18**	USFS	TreesCount! 2015	2012	6	2,200
**19**	USFWS	Urban Edge Habitat Use	2015	3	
**20**	USGS	Cactus Moth Detection & Monitoring	2004	14	

**Fig 1:**
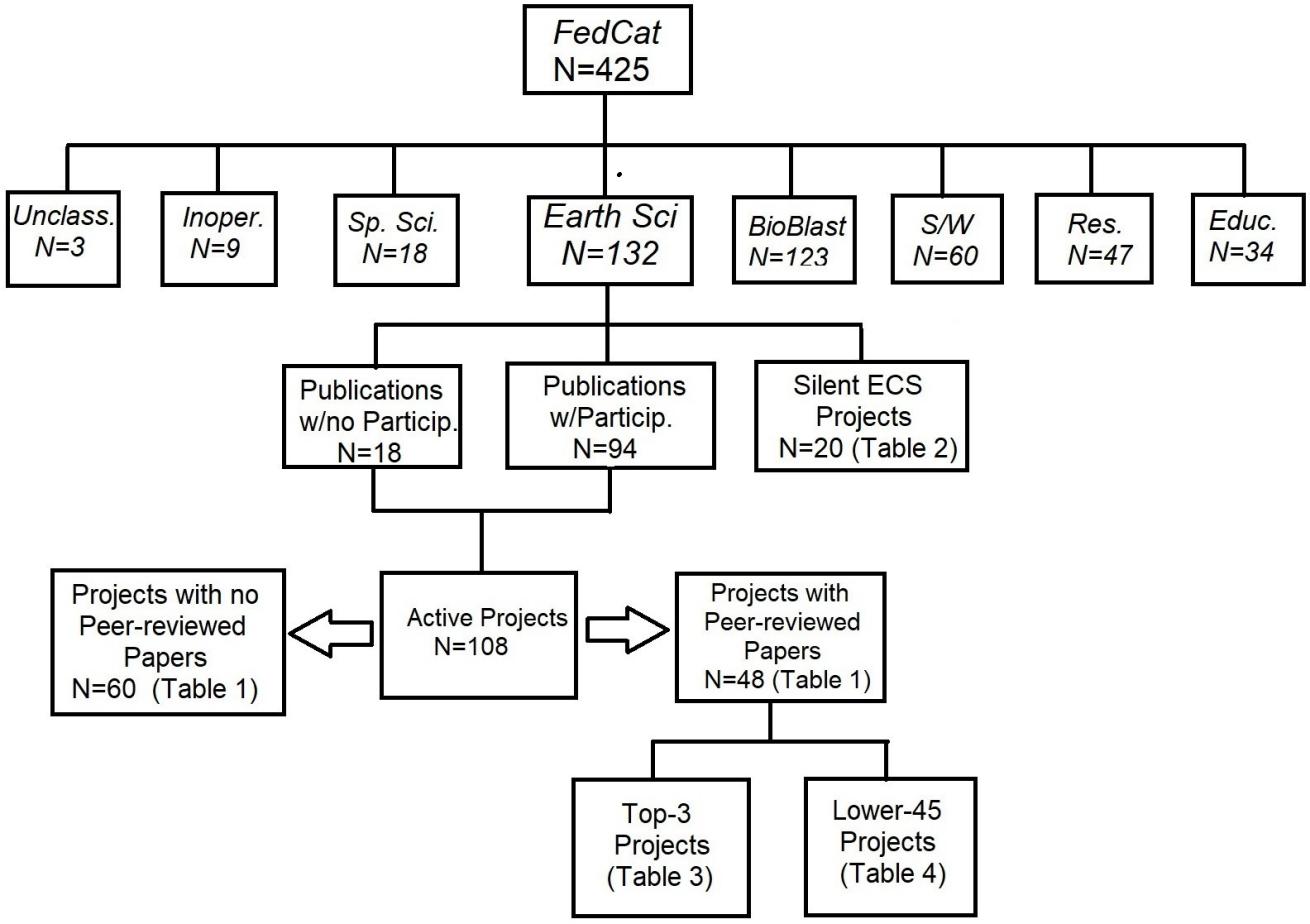
Numerical breakdown of the CS projects used in this study.

For convenience, Fig 1 shows the complete breakdown of how the various samples were created starting from the FedCats archive.

The citation frequency for each of the 782 peer-reviewed papers was extracted from the online data base provided by WoS using the standard search tools provided on the website. On the Advanced Search page, I entered the Boolean string for the first-author’s last name and the journal. I then selected the year range spanning the year of the article publication. The search provided a page of ‘hits’ and from these I selected the specific article by the author being studied. A Citation Report was then produced by WoS for that article, which included the total number of citations accumulated from the time of the publication to the end of 2018, which is the end year of the current study.

In the majority of the cases, it was only necessary to search under the first-author’s surname and initials for the year of the publication. The aggregated results for the citations of all peer-reviewed papers published for a given project are provided in Table 3 (column 8) for the Top-3 projects and Table 4 (column 10) for the Lower-45 projects. The projects are ordered by their inception year in column 4. In column 7 the median number of authors per paper is provided. In Table 3, we also include the three largest SSCS projects identified by [5] in order to compare the Top-3 projects between earth science and space science. In Table 4, the projects have been partitioned into five groups based upon their inception dates in order to subsequently analyze changes between projects within approximately the same age groups. Columns 9 and 10 indicate the peer-reviewed papers per year (hereafter: PPY) and citations per peer-reviewed paper (e.g. the Impact Factor, IF) generated by each project.

**Table 3:** Summary for Top-3 SSCS and ESCS projects.

(1)	(2)	(3)	(4)	(5)	(6)	(7)	(8)	(9)	(10)
Project	Log(N)	Year	Age	Total	Ref.	Auth.	Cites	PPY	IF
Christmas Bird Count	4.9	1900	119	349	306	1	3896	2.6	13
The GLOBE Program	5.1	1994	25	608	61	3	1874	2.4	31
eBird	5.6	2003	16	240	196	4	2084	12.3	11
Galaxy Zoo	5.4	2008	10	58	57	12	2984	5.7	52
Einstein@Home	5.7	2008	10	22	22	20	384	2.2	17
Planet Hunters	5.5	2010	6	12	12	21	291	2.0	24

**Table 4:** Summary for the Lower-45 ESCS projects by age cohort.

(1)	(2)	(3)	(4)	(5)	(6)	(7)	(8)	(9)	(10)	(11)	(12)
	Agency	Project	Year	Age	Log(N)	Total	Ref.	Auth.	Cites	PPY	IF
1	NPS	Woodcock Survey	1968	51	2.8	29	13	2	322	0.3	25
2	NOAA	SKYWARN	1975	44	5.6	12	2	3	108	0.0	54
3	NPS	Raptors	1983	36	3.1	17	8	1	158	0.2	20
4	EPA	Lakes of Missouri	1992	27	2.0	2	2	4	10	0.1	5
5	NOAA	Beach Watch	1993	26	2.0	25	5	15	44	0.2	9
6	EPA	The Secchi Dip-In	1994	25	3.5	8	2	4	21	0.1	11
7	USGS	NA Amphibians	1997	22	3.0	11	10	6	197	0.5	20
8	NOAA	CoCoRaH	1998	21	4.3	31	26	4	163	1.2	6
9	NOAA	WormWatch	1999	20	3.0	29	19	3	1475	1.0	78
10	NOAA	CWOP	2000	19	4.5	4	1	2	0	0.1	0
11	USGS	Did You Feel It?	2003	16	6.3	11	8	3	306	0.5	38
12	NOAA	COSS	2005	14	3.0	24	15	7	170	1.1	11
13	NPS	Loon Project	2005	14	3.3	12	6	3	24	0.4	4
14	USGS	SEANET	2006	13	1.7	5	1	3	0	0.1	0
15	NSF	Project BudBurst	2007	12	3.5	11	10	2	304	0.8	30
16	USGS	Quake Catchers	2008	11	3.4	10	10	6	189	0.9	19
17	EPA	Gardenroots	2008	11	2.0	3	3	5	96	0.3	32
18	NPS	Cascade Butterflies	2008	11	2.3	4	1	2	0	0.1	0
19	NASA	GLOBE at Night	2009	10	4.3	68	7	5	40	0.7	6
20	USFS	Gros Ventre Project	2009	10	1.4	1	1	1	0	0.1	0
21	USGS	Bald Cypress Net	2010	9	2.0	4	1	2	4	0.1	4
22	USFWS	Alaska Bats	2011	8	2.0	8	2	4	0	0.3	0
23	USGS	Michigan AMBLE	2011	8	1.6	9	2	5	3	0.3	2
24	NSF	WiEye	2011	8	4.8	23	2	3	1	0.3	1
25	USDA	CARM	2012	7	3.0	5	2	8	0	0.3	0
26	NOAA	Cyclone Center	2012	7	3.6	6	1	3	11	0.3	0
27	USGS	Insect Monitor	2012	7	2.4	22	2	6	1	0.3	1
28	NOAA	Marine DMA	2012	7	2.7	8	2	3	16	0.3	0
29	NOAA	ISeeChange	2012	7	3.7	10	1	3	1	0.1	1
30	EPA	LEOnet	2012	7	2.3	5	1	7	0	0.1	0
31	NOAA	Old Weather	2012	7	4.3	6	1	4	0	0.1	0
32	USFS	Backyard Bark Beetles	2013	6	2.8	8	6	4	8	1.0	1
33	NIH	EyeWire	2013	6	4.9	8	1	4	5	0.2	5
34	EPA	Adopt-a-Stream	2013	6	2.0	4	1	2	0	0.2	0
35	NSF	Notes from Nature	2013	6	3.8	5	1	16	28	0.2	28
36	USDA	Snow Surveyors	2014	5	2.0	2	26	3	642	5.2	0
37	NIH	Barcode Long Island	2014	5	3.8	2	2	39	0	0.4	0
38	NSF	Foldit Game	2015	4	4.8	8	5	2	49	1.3	10
39	USGS	CyanoTRACKER	2015	4	2.0	8	1	3	3	0.3	3
40	NPS	Dragonfly Mercury	2015	4	3.6	10	1	2	0	0.3	0
41	NSF	Season Spotter	2015	4	4.1	1	1	6	1	0.3	1
42	NASA	MAPPD	2016	3	2.7	4	2	6	7	0.7	4
43	NSF	Evolution Project	2016	3	3.1	3	2	20	2	0.7	1
44	NOAA	Hui o ka Wai Ola	2016	3	1.4	3	1	6	4	0.3	4
45	NASA	Landslide Reporter	2018	1	1.6	2	2	4	113	2.0	57

## 4.0 Analysis

### 4.1 Productivity gauged by published papers

By this measure, the Top-3 projects shown in Table 3 accounted for 72% of the total earth science publications, with a median of 196 papers per project (PPP). When adjustment is made for the age of the project from inception to the end of 2018, the median rate of paper production for the Top-3 projects is 2.6 papers/year (PPY). The Lower-45 projects in Table 4 produced the remaining 219 papers for a collective, median productivity of 0.3 PPY. Over half (52%) of the projects have only been in existence since 2010 and show the effect of younger projects having a significantly lower median productivity (col. 8) than the older projects. The conclusion is that the PPY for the Lower-45 projects is eight-fold lower than what was determined for the Top-3 producers in Table 3.

For the typical projects of Table 4, the Lower-45 cohort summary in Table 5 reveals that there is little change in the median number of authors per paper (APP: col. 2) over the post-1990 period, though slightly fewer seem indicated by the 1900–1992 cohort. This trend is limited in confidence by the small-number statistics of the earliest projects, which account for only 7% of the projects in the sample. Since the number of authors per paper is virtually the same between the Top-3 (APP = 3) and the Lower-45 (APP = 4) projects, the difference in paper output P, and output rates PPY cannot be explained by one project having numerically more scientists participating in the research. This might suggest that a very small number of authors, perhaps one or two, are the primary source of the project output and are actively involved in reporting and promoting the scientific results.

**Table 5:** Lower-45 project summaries by age cohort.

(1)	(2)	(3)	(4)	(5)	(6)	(7)
Group	Median	Median	%	%	Median	Median
	Authors	Age	Projects	Papers	PPP	PPY
1900–1991	2	44	7	11	8	0.3
1992–1999	4	22	13	29	10	0.5
2000–2008	3	12	24	29	3	0.4
2009–2012	4	7	24	8	2	0.3
2013–2018	4	4	31	24	1	0.3

Although the Top-3 projects had a median of 100,000 participants (Table 3 col. 2), the Lower-45’s median of 1,000 indicates that there may be some relationship between the participatory scale of the project and its productivity. However, two projects, *SKYWARN* and *Did you feel it*?, have participation levels comparable to those of the Top-3 projects yet produced significantly less output, so among this second group of projects, the level of public participation is in detail not a good predictor of the project’s productivity.

**Fig 2:**
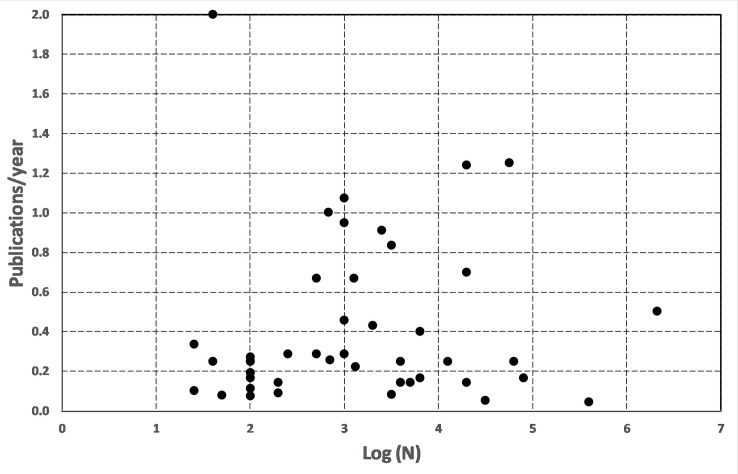
Peer-reviewed Publications per Year (PPY) for each of the 44 projects compared to the estimated number, N, of participants. The exceptional Lower-45 project *Snow Surveyors* (Log(N) = 2.0 and PPY = 5.3) has not been plotted so that the distribution details of the remaining projects can be better seen.

Table 5 also summarizes the median number of papers per project (PPP col. 6) in each age cohort. Although the list of published, peer-reviewed papers should be complete through 2018, nevertheless, there is a marked decrease from 8–10 PPP to 1–3 PPP at about the year 2000, representing an apparent four-fold decline in published papers among the newer projects. One possible explanation for may be that the older projects involved the still-novel CS approach to well-defined and intuitively comprehensible scientific goals. By contrast, the modern projects no longer seem novel, and their topics are perhaps less understandable to the CS volunteering community. A comparison of the publication rate with the participatory scale of the Lower-45 projects is shown in Fig 2. The large dispersion in PPY for values of Log(N) along with the flat distribution in PPY yields a linear regression with a slope of 0.0 and R^2^ < 0.5, which indicates no correlation and that 99.5% of the variation is caused by random error. The two outlier points at PPY = 2.0 (*Landslide Reporter*) and 5.3 (*Snow Surveyors*) are exceptional given the small number of participants.

The conclusion from this study is that, as for the SSCS projects studied by [5], the ESCS projects show as one might expect a strong distinction between the Top-3 and most productive projects and the much larger Lower-45 population of projects that also have significantly lower participation levels. There is, however, little difference in the median number of authors between these two groups. The participation level and scale of the project apparently has an impact on the publication rates of the projects with the larger projects in the Top-3 group generating about eight-fold higher PPY rates than the more numerous and smaller-scale projects in the Lower-45 group. For project performance in terms of publication output and annual output rates, it is distinctly more advantageous to be a project with over ca 100,000 participants.

### 4.2 Productivity gauged by the citation index: CI

**Fig 3:**
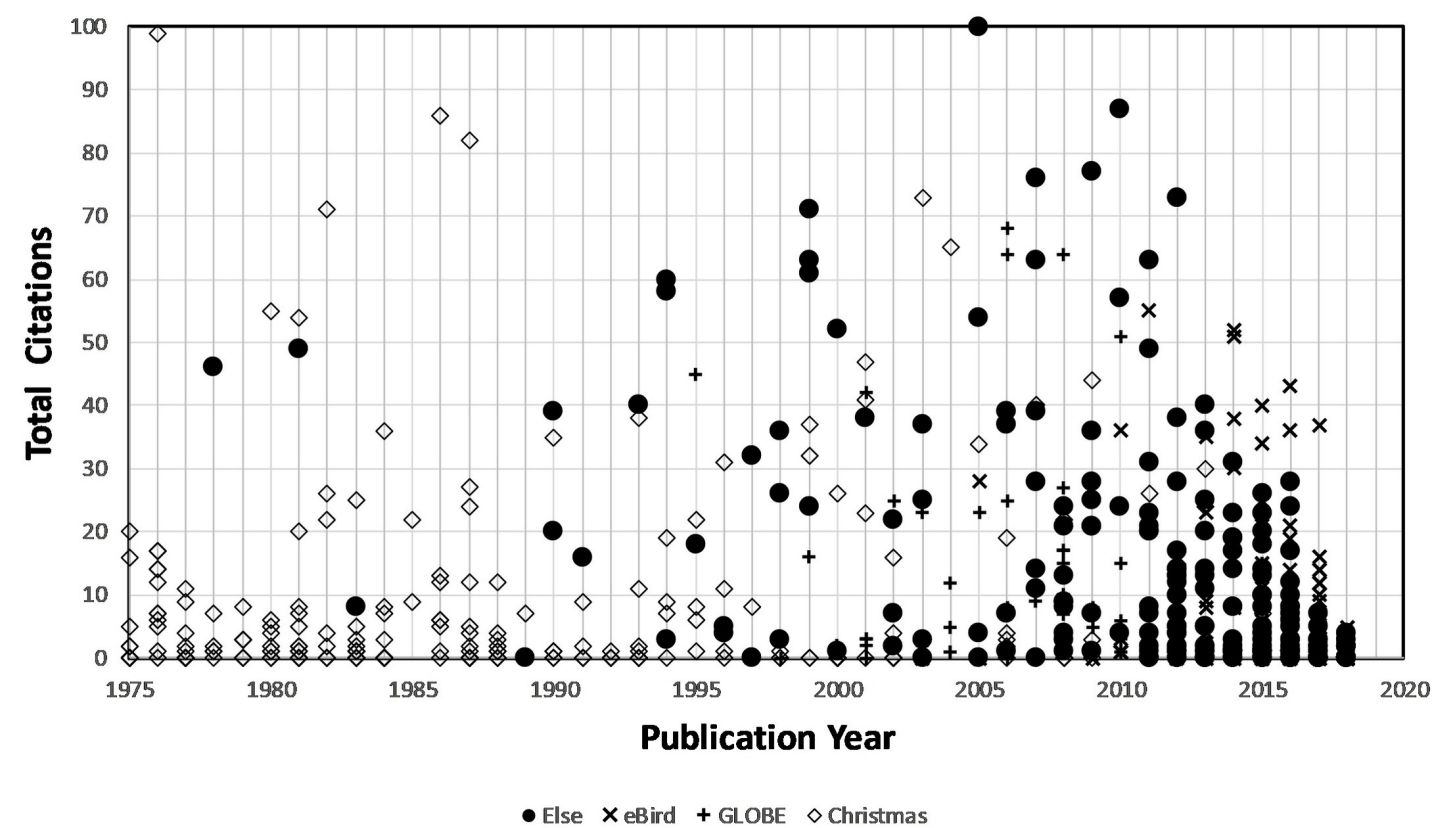
Total citation counts as of 2018 by publication year since 1975 for peer-reviewed papers for the Lower-45 projects (filled dot), and the Top-3 projects *eBird* (x), *GLOBE* (+) and *Christmas Bird Count* (diamond).

The cumulative citations for each of the 782 papers in this study were identified using the WoS online citation catalog. The full ensemble of 48 projects with peer-reviewed papers generated a total of 12,380 citations, with the specific sub-totals by the Top-3 projects shown in Table 3 (col. 8) contributing 7,854 citations and the Lower-45 shown in Table 4 (col. 10) contributing 4,526 citations. Clearly, the Top-3 projects out-performed the Lower-45 projects using the gross number of citations as a measure. It is clearly beneficial to have projects that produce large numbers of papers in order to generate the largest numbers of citations. I also expect that the more recent papers will be numerically disadvantaged in the citation count and this is borne out by Fig 3, which shows the publication year of each paper and its cumulative citations.

Although the citations generally increase for both groups through ca 2015, there is a progressive fall-off after ca 2015. Since it takes up to five years or longer before a paper begins to be cited, this lag probably explains the 2015–2018 fall off. These distributions are, however, generally what would be expected since, all other factors being equal, older papers published before ca 2010 ought to have accumulated more citations than more recent ones. Fig 3 is a snapshot of how the project papers compare as of the end of 2018 when the citation counts were completed. In time, the points will shift upwards as more citations accumulate after 2018. Nevertheless, Fig 3 gives a rough comparison of how papers published by the various projects stand in relation to each other. What is seen is that for most years from 1975–2010 the papers from the Lower-45 and Top-3 are well-mixed during any given year, which indicates that papers from diverse projects are being cited about equally well irrespective of whether they are in the Top-3 or Lower-45 projects. After 2010, and especially after 2012 there is a sharp decline for all groups in the citations for the Lower-45 papers but not for the *eBird* project, which continues to generate papers between 2010–2018 that are producing significant numbers of citations. This suggests that there are either fewer papers being published by the Lower-45 cohort after 2010, or those papers that are published are being under-cited. The former possibility can be discounted because in Table 5 there was no decline in PPY for this group during this time interval. This suggests that more recent papers among these projects are not being cited at the same rate as papers in this cohort before ca 2010.

### 4.3 Papers with no citations

According to [34], a study of 12,000 journals in the WoS database, as many as 10% of published articles are never cited. Earlier studies by [35] found non-citation rates for peer-reviewed papers between 4–11% for papers in the natural sciences with rates as high as 65% in the humanities.

The ensemble of 783 peer-reviewed papers included 266 that were not cited by the end of 2018. Given that the publication record ends in 2018, if we apply a grace period of two years before we expect the first citations to appear, we find that papers published in 2017 and 2018 account for 84 of these cases and have not as yet begun to have citations to them. Correcting the percentages for the four project categories, the zero-citation rates are Else = 12%, eBird = 14%, GLOBE = 18% and Christmas = 0%. While these no-citation rates of 12–18% appear high, the study by [35] suggests that these rates may not be unusual, and moreover should be interpreted with caution.

According to [36], “It is a sobering fact that some 90% of articles that have been published in academic journals are never cited. Indeed, as many as 50% of papers are never read by anyone other than their authors, referees, and journal editors.” Nevertheless, according to [37] some uncited research provides important information for databases that other investigators rely upon. In other instances, uncited research in online, peer-reviewed journals such as PLOS One may nevertheless be viewed and downloaded thousands of times. An example of this is a paper by [38], which [34] identifies as having never been cited but has been viewed via social media over 1,500 times and downloaded 500 times. These uncited-but-popular papers can become part of the underlying sea of uncited but influential literature that all researchers share, and which new online citation indices called Altmetric Attention Scores [39,40] attempt to capture.

### 4.4 A general comparison with non-CS earth science research papers

Ultimately, the goal of this investigation is to compare ESCS projects against non-CS research approaches to assess whether ESCS projects are as productive based upon standard bibliographic indices as conventional research approaches. This comparison was performed for space science projects by [5] who found that CS projects perform as well, and by some measures, even better than conventional papers using non-CS approaches. The question is whether the same conclusion applies to earth science CS projects. The ESCS projects in Tables 3 and 4 cover primarily the biological sciences rather than geophysics, so a survey of the biological publications in the earth sciences (ecology, bio statistics, wildlife management, ornithology, etc) would appear to be a good place to start.

In the previous study, the non-CS, space science papers used as a reference appeared in only three main journals: *The Astronomical Journal*, the *Monthly Notices of the Royal Astronomical Society* and *The Astrophysical Journal*. The previous strategy was to identify all papers published in these three journals for the year 2000, and then follow the collective history of the citations for these papers through 2018. In contrast, the ESCS papers were published in over 352 different journals whose audiences are specialized, and with none accounting for more than about 10% of the peer-reviewed publications in this study. Among the Top-3 programs, which are primarily in the field of ornithology, the most popular journals were *Ecology* and *Biological Conservation*. The most popular journals for the Lower-45 papers were *Frontiers in Ecology and the Environment*, *Global Change Biology*, and the *International Journal of Biometeorology*. Similarly to the previous space science study, we selected a reference year, in this case 2003, because this was the first year of publication for the *Frontiers in Ecology and the Environment*, and it represents the start year of the majority (80%) of the ESCS projects. The counting via WoS was performed over the interval 2003–2018. A total of 980 papers were identified in these five journals and yielded 78,575 citations over the 15-year period.

**Fig 4:**
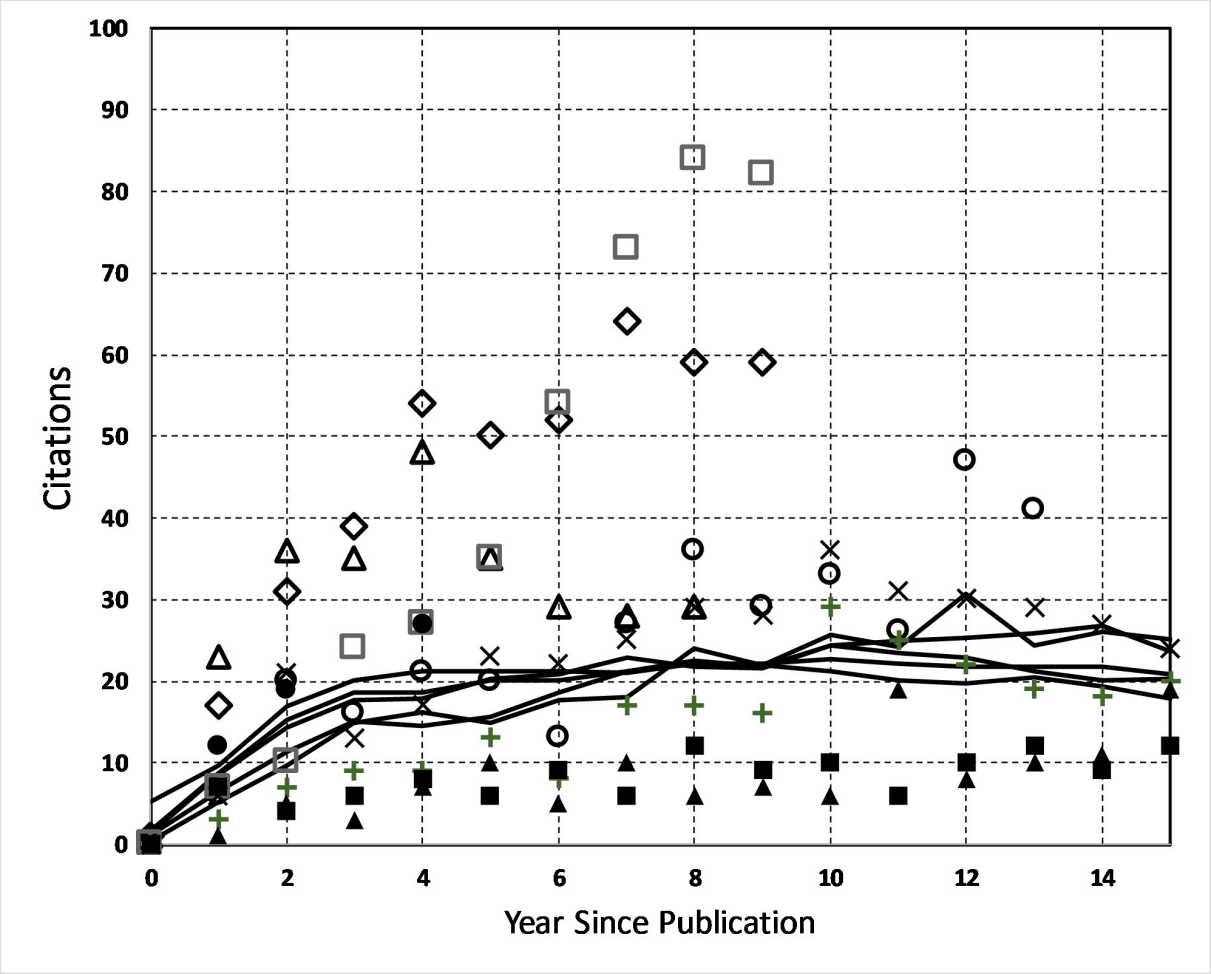
Citation history for the Top-7 ESCS papers with more than 257 citations compared to the normalized and re-scaled journal citation histories (solid lines). Bonney [42](square), Penuelas [43](X), Reich [44](circle), Root [45](filled square), Verner [46](filled triangle), Morys [47](+) and Buckley [48] (triangle). Also shown are the two top performers in the SSCS sample: Lintott [49](diamond) and Willett [50](filled circle).

The annual citation history of these non-CS, earth science papers has been normalized by dividing each journal’s citations by the total number during the 2003–2018 reference interval. The resulting normalized history was then multiplied by an arbitrary factor of 300 to place the curves on the same scale as the ESCS papers. The resulting hypothetical non-CS citation history curves for each journal are shown in Fig 4. The normalization and re-scaling was performed so that these journals can be used as proxies to represent the typical citation history profiles for the aggregate papers published in them, and to place them in the same range of annual citations as found for the ESCS papers with the highest citations. According to [41], after five years, one paper in 1,000 had reached 257 cumulative citations so we can compare the ESCS papers with > 256 citations against this earlier study. These ‘Top-7 ESCS’ papers were: [42] with 464 citations, [43] from the *GLOBE* program with 422 citations, [44] with 336 citations, [45] from the *Christmas Bird Count* project with 320 citations, [46] also from the *Christmas Bird Count* project with 303 citations, [47] from the *GLOBE* project with 277 citations, and finally [48] with 270 citations. Also included for comparison are the two popular SSCS papers by [49] with 427 citations and [50] with 268 citations.

What is seen in Fig 4 is that the profiles of the most-cited ESCS papers do follow the history trend of the average non-ES papers (solid lines) with annual citations that gradually increase between 1–5 years after publication, and then level off with a long sustained annual citation rate lasting in some instances 15 years. What is noteworthy is that the ESCS paper by Bonney[42] and the SSCS paper by Lintott [49] continue to increase their annual citations until about Year-8 at which point they appear to level-off. Since for Lintott Year-8 occurs in 2016, and for Bonney it is 2017, these years occur before the citation count limit of 2018 and so the leveling-off may be a real feature of the citation history. At least the most-cited ESCS papers behave very similarly to non-CS papers with a peak at about 1–5 years after publication. The most-successful of these enjoy a more prolonged growth to Year-8 before they, too, appear to reach a plateau.

### 4.5 Impact factor

The Impact Factor is a ratio of the number of citations over a specific span of time divided by the number of papers published over the same time interval. In the case of CS projects, the appropriate time interval is the difference between the inception year of the project and 2018 when this study was concluded. For example in Table 3, The GLOBE project had 61 peer-reviewed papers that garnered 1874 citations resulting in an IF = 1874/61 = 31. In Table 3 (col. 10) and Table 4 (col. 12) the IF was calculated for each project and summarized in Table 6 along with data on the SSCS projects provided by [5]. For example, the Lower-45 ESCS projects with 219 papers and 4,526 citations (Table 4) yields an aggregate IF of 21. By this measure, the comparisons suggest that the Top-3 space science projects tend to have higher impacts than the corresponding earth science projects, but that the general populations of these projects excluding the Top-3 favor the earth science projects in impact.

**Table 6:** A comparison of impact factors for citizen science projects.

(1)	(2)
	IF
**Top-3**	
SCS-*Galaxy Zoo*	52
SCS-*Planet Hunters*	24
SCS-*Einstein@Home*	17
ECS-*The GLOBE*	31
ECS-*Christmas Bird Count*	13
ECS-*eBird*	11
**Lower**	
SSCS(N = 23)	10
ESCS(N = 45)	21

A study by [51] has investigated the citation rates for the 500 most-cited papers across 236 scientific categories, and found that in general these papers achieve IF> 45. In the comparison with non-CS papers described in Section 4.4, the 980 papers with their 78,575 citations yielded an IF = 78575/980 = 80 among the most popular two or three journals out of 352 in the SSCS sample, but implies that the ESCS papers fall well-below the average natural science IF. Only three of the Top-3 projects equal or exceed this level of impact described by [51], and are significantly below the IF computed for the reference papers published in 2003. However, studies of the more numerous papers indicate that significantly lower IFs are, in fact, more common. For instance, according to [52] between 1990 and 2010, natural science papers had a typical IF of about 25 with significant variations within the sub-disciplines. We see in Table 6 that the Top-3 projects cluster around IF = 25, with the ESCS projects generally in the lower-half of the range. Is this difference significant?

Although comparing CS with non-CS papers is problematical because of the different methodologies employed to gather and analyze data, we can investigate whether CS projects in two distinct disciplines have similar impacts. A detailed comparison between the IF distributions for the Lower-45 ESCS and Lower-23 SSCS projects can determine whether these projects using similar citizen science approaches also have similar impacts.

**Fig 5:**
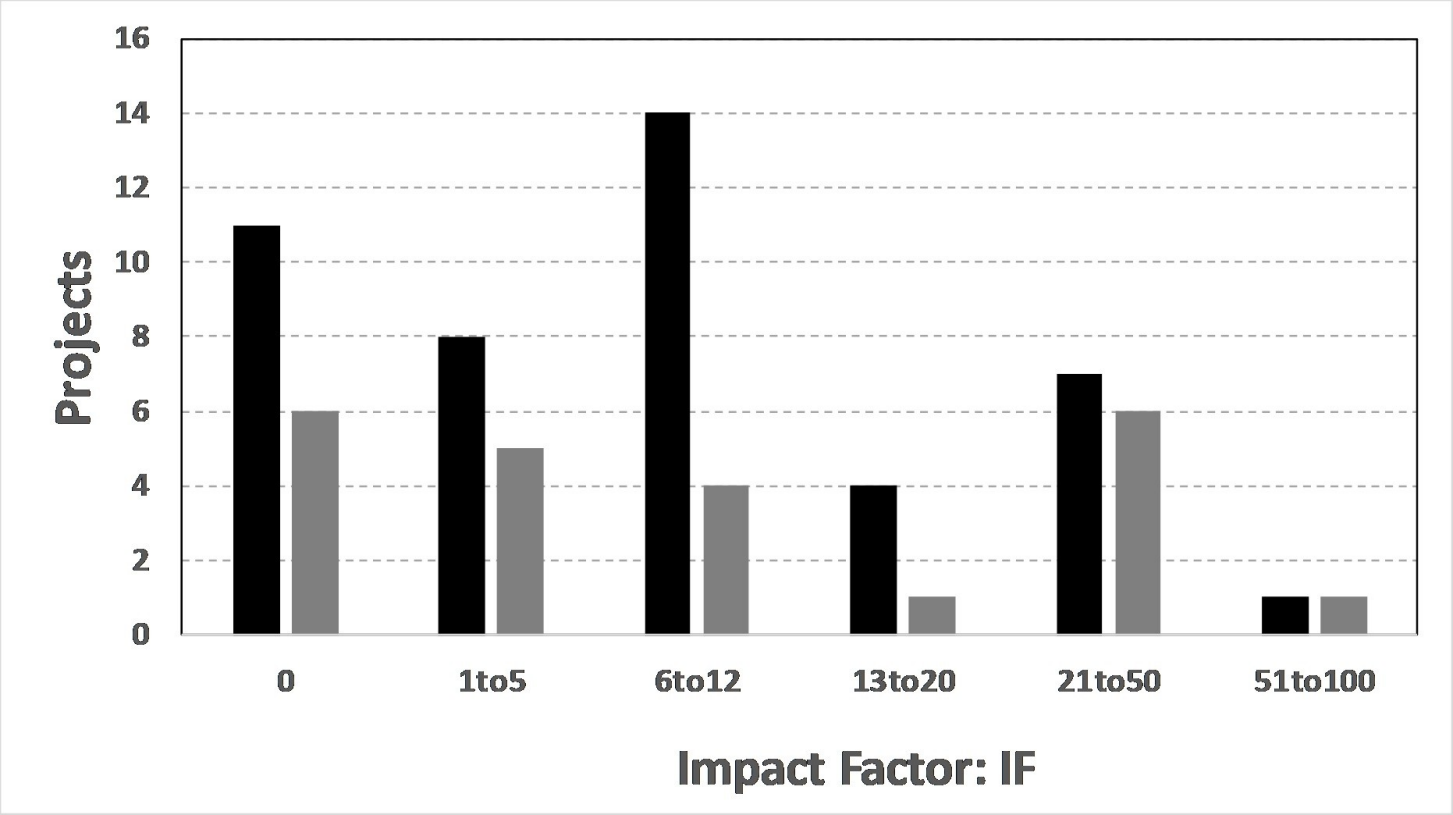
Histogram of CS project impact factors for the Lower-45 ESCS projects (black bars) from this study, and 23 SSCS projects (gray columns) based upon [5].

We can check the null hypothesis that there is no significant difference between the ES and SS groups by performing a one-sided, Student’s T-test using the N = 6 categories in Fig 5. The total sum of the differences ∑D = 22 and the sum of the squared differences ∑D^2^ = 144. The T-score is computed from the formula

$$T=\frac{(\sum D)/N}{\sqrt{\frac{\sum D^{2}-(\frac{{(\sum D)}^{2}}{N})}{(N-1)N}}}$$

This results in T = (22/6) / [(144–484/6)/(5x6)]^1/2^ = 2.52 for 5 degrees of freedom. The corresponding T-value is 2.05 for rejecting the null hypothesis at the 5%-level (i.e. accepting the null hypothesis at the 95%-level). Since the T-value is greater than the calculated 2.52, the SSCS and ESCS IF distributions are, therefore, statistically different. Judging from the histograms in Fig 5, most of this difference comes from the IF values between 6 and 12. One can conclude that the more numerous citizen science projects in these two areas have statistically significant differences in impact with the earth science projects performing somewhat better than the space science projects as evidenced by Fig 5.

### 4.6 The H and M-indices

For the ensemble of 48 ESCS projects, the combined, ranked project citations in Tables 3 and 4 yield H = 23. The CS projects in both earth and space science achieve aggregated H-indices that suggest they are as impactful as the publication efforts of equivalent individual scientists applying for a tenure decision. Generally, only a small number of citizen science projects have H-indices that place them above the threshold for ‘tenure’ (H = 18) in earth science: *Christmas Bird Count (H = 31)*, *eBird (H = 23)*, and *The GLOBE (H = 17)*. In space science the corresponding projects are *Einstein@Home*, *Planet Hunters* and *Galaxy Zoo*. The large dispersion in H-indices especially for the most recent years since 1990, indicates that there is no significant correlation with project age after 1990. The ESCS and SSCS groups each yield a flat regression (slope ±0.05) with R^2^ <0.005, which indicates that no correlated behavior exists in this data to a significance exceeding 99%. However as expected, the projects with the largest number of publications, at least within the limitations of small-number statistics, tend to have the highest H-indices.

**Fig 6:**
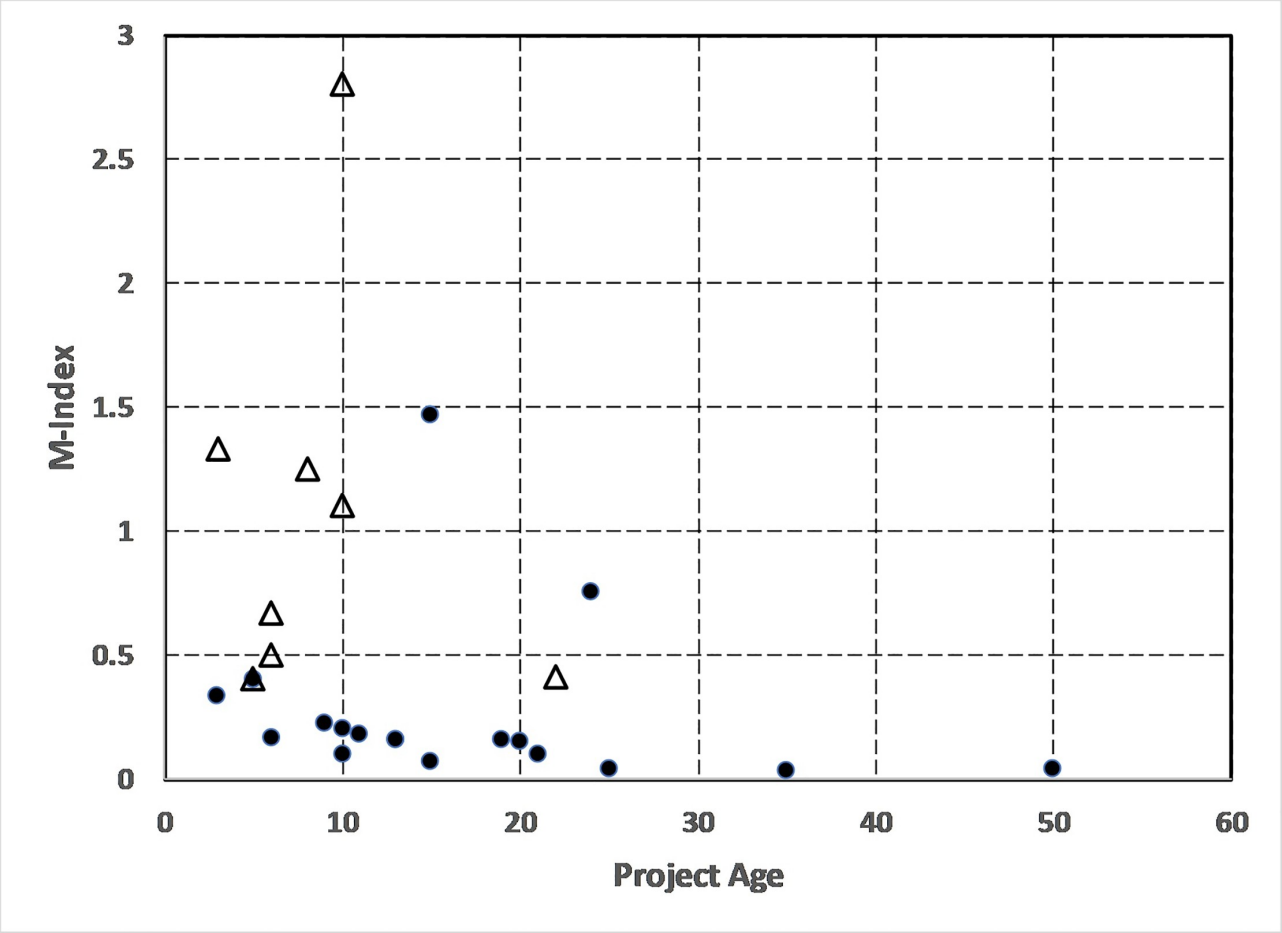
Aggregated M-index for projects with more than three publications in SSCS (triangles) and ESCS (dots) with *Christmas Bird Count* (age 118 years and M = 0.26) excluded.

The age-corrected M-index has been suggested by [28] as a better measure of a scientist’s publication impact because it accounts for the number of years that publications have been in print. In the case of this investigation, it would be the age of the CS project that would be the best time-base for normalization so that younger projects are not disadvantaged compared to older CS projects. This measure was implemented by simply dividing the H-index by the total years of operation of each project resulting in Fig 6. As a comparison of the SSCS and ESCS projects, the M-index calculated in this way shows that the younger space science projects have noticeably larger M-indices than the earth science CS projects. Because the comparison is between one category of citizen science projects against another, the comparison is straight-forward because both groups essentially use the same methodology of crowdsourcing their data analysis, and we are using the same ‘project age’ time base for the normalization. Since the project age-effect has been normalized-out, the remaining variation suggests that the SSCS projects may be more impactful than the ESCS projects.

According to an assessment of the M-index by [19], M = 1 (e.g. H = 20 after 20 years of research) is the threshold for a ‘successful’ scientist, while M = 2 is the threshold for ‘outstanding scientists likely to be found only at the top universities or major research laboratories’. By this measure, virtually all of the ESCS projects fall below the M = 1.0 threshold for a ‘successful’ scientist while half of the SSCS projects fall above this threshold.

### 4.7 Other measures

Another measure of impact proposed by [53] is the number of papers that exceeded a specific number of citations by the end of a survey year. From their study, among the 58 million papers indexed by the *WoS*, only 14,500 papers (three-in-1,000) achieved more than 1000 citations by 2014. For space science papers in particular [41] found that after five years, one paper in 1,000 had reached 257 cumulative citations. For the SSCS study [5], an equivalent of 26 papers per 1000 were found to have reached the same level of citations after five years. The conclusion by [5] was that the SSCS papers had higher odds of being very successful papers than non-SSCS papers by this measure.

A similar analysis was performed for all 48 ESCS projects, which resulted in five papers having more than 257 citations after five years, which I call the P257-index, for an equivalent of 10 papers per 1000. Taken separately, the Top-3 papers had a rate of 5 in 1000 (*eBird*); 33 in 1000 (*GLOBE*); and 7 in 1000 (*Christmas Bird Count*), while the Lower-45 had a rate of 10 in 1000. These rates are substantially higher than that found by [41] at least for space science papers in general.

Assessing the corresponding rates for non-ESCS papers is difficult. For earth science papers, research papers appear in over 137 different journals. The most popular of these are listed in Table 7 and represent 18% of all cited ESCS papers. To perform the citation study I selected the median year for the ESCS papers, 2011, and identified the papers published in that year for each journal. The WoS service provides a means for quickly determining the total citations and statistics for the ensemble of papers using the ‘Citation Report’ feature, resulting in a total of 382 papers (col. 4) out of 5,890 in the sample achieving more than 257 citations after 5 years. The equivalent rate is then estimated to be 65 papers per 1000. The journal Nature is an outlier, so if we only consider the remaining non-CS papers the totals are 3,224 papers, with 42 papers exceeding 257 cites for an equivalent of 13 per 1000.

**Table 7:** Estimated statistics for non-ESCS papers published in 2011.

(1)	(2)	(3)	(4)	(5)	(6)
Journal	Non-CS Papers	CI	P > 257	IF	H
Biological Conservation	293	10,814	2	37	52
Ecology	294	13,605	3	46	59
Wilson Bulletin	122	673	0	6	12
Frontiers in Ecology and the Environment	192	5,256	1	13	41
Global Change Biology	310	21,276	9	69	79
International Journal of Biometeorology	116	3,369	2	29	30
Ecological Applications (2010)	177	6,450	1	36	47
Methods in Ecology and Evolution	126	11,754	12	93	49
Conservation Biology	134	4,544	2	34	39
Biological Invasions	212	4,753	0	22	34
Ecology Letters	167	15,236	7	91	69
Ecology and Evolution	262	5,183	0	20	34
Nature	2,666	323,549	340	121	292
American Naturalist	163	5,166	0	32	41
Climate Research	83	2,204	1	27	24
Journal of Biogeography	195	7,542	2	39	46
Agricultural and Forest Meteorology	186	6,353	0	34	44
Journal of Wildlife Management	192	3,051	0	16	28
**Totals:**	5,890	450,778	382	<16>	<18>

The IF and H-indices in the last row are shown in brackets are the median of the values in column 5 and 6.

The results of all of the findings in this study are summarized in Table 8. The papers per year (PPY: col. 2) and the median number of authors per paper (APP: col. 3) are obtained from Tables 3 and 4. The percentage of papers with no citations (col, 4) is taken from the discussion in Section 4.3. The median IF (col. 5) is from Table 6. The median H-index and M-index are obtained from the discussion in Section 4.6, and the P257 index is from Section 4.6.

**Table 8:** Comparisons of various indices of citizen science project impact.

(1)	(2)	(3)	(4)	(5)	(6)	(7)	(8)
**Source**	**PPY**	**APP**	**C0**	**IF**	**H**	**M**	**P257**
**ESCS**							
Top-3	2.6	3	12%	13	23	0.75	15
Lower-45	0.3	4	14%	21	23	0.16	10
**SSCS**							
Top-3	0.11	14	3%	24	11	1.7	33
Lower-23	0.06	11	10%	10	4	0.5	0

The comparison between the two groups of citizen science research indicates that the earth science projects produce more papers per year with fewer authors than the space science citizen science projects, but have slightly higher rates of papers with no citations. The other indices lead to relatively mixed assessments in which the Top-3 projects from each subject area have higher M-values and odds of being in the Top-1000 papers for space science. The more numerous projects, however are higher performers in earth science than space science in terms of the H index and odds of being in the Top-1000 papers.

## 5.0 Conclusion

### Publications

A total of 783 peer-reviewed citizen science research papers in the area of earth science were identified through the end of 2018. The Top-3 projects were *Christmas Bird Count* (306), *eBird* (196), and *The GLOBE Project* (61), which collectively account for 563 papers (72%). The second ‘Lower-45’ group, contributed the remaining 219 papers. Although 80% of the projects have only been in existence since 2000, the Top-3 projects have a nearly ten-fold higher rate of paper production (2.6 papers per year) compared to the Lower-45 projects. Although the difference cannot be attributed to the median number of authors per paper in each group, which is nearly the same (e.g. 3–4) for each group. The Top-3 projects have a median of nearly 100 times the number of participants than the Lower-45 projects, which suggests a higher throughput of data from which papers could be generated. However, within the Lower-45 group, there is no commensurate correlation between annual paper output and participatory scale, although these projects tend to be significantly younger than the Top-3 projects.

### Citations

The full ensemble of 48 projects with peer-reviewed papers generated a total of 12,380 citations by the end of 2018, with the Top-3 projects contributing 7,854 citations and the Lower-45 with 4,426 citations. In terms of annual paper citations, for most years between 1975–2010 the papers from the Lower-45 and Top-3 are well-mixed during any given year, which indicates that papers from diverse projects are being cited about equally. After 2010, and especially after 2012 there is a sharp decline for all groups in the citations for the Lower-45 papers but not for the Top-3 projects, which continue to generate papers between 2010–2017 that are producing significant numbers of citations. This suggests that there are either fewer papers being published by the Lower-45 cohort after 2010, or those papers that are published are being under-cited. The former possibility can be discounted because there was no decline in papers-per-year for this group during this time interval. This suggests that more recent papers among these projects are not being cited at the same rate as papers in this cohort before ca 2010. This is supported by the discovery that in terms of the number of papers from each project that are not being cited at all, the Lower-45 rate of 12% is comparable to the median rate of 14% for the Top-3 projects.

When the annual citation histories of the earth science projects are compared to non-citizen science projects in earth science, the profiles of the most-cited CS papers do follow the history trend of the average non-ES papers with annual citations that gradually increase between 1–5 years after publication, and then level off with a long sustained annual citation rate lasting in some instances 15 years. The most-successful of these projects enjoy a more prolonged growth to Year-8 before they, too, appear to reach a plateau.

### Impact factors

A comparison of the earth science and space science citizen science projects which use similar data gathering and analysis methodologies suggests that the Top-3 space science projects tend to have higher impacts (IF = 24) than the corresponding earth science projects (IF = 13), but that the general populations of these projects excluding the Top-3 favor the earth science projects (IF = 21) over the space science projects (IF = 10) in impact. Studies by other investigators of natural science research papers between 1990 and 2010 find that typical impact factors near IF_n_ = 25 are common with considerable sub-discipline variation. Both the earth and space science citizen science papers fall below IF_n_, and only three of the Top-3 projects equal or exceed this level of impact.

### H and M-indices

The CS projects in both earth and space science achieve aggregated H-indices that suggest they are as impactful as the publication efforts of equivalent individual scientists applying for a tenure decision. Generally, only the Top-3 citizen science projects in earth and space science have H-indices that place them above the threshold for ‘tenure’. The H-index is known to be flawed in that it is age-dependent favoring scientists and programs that have longer publication histories. The M-index is created by dividing the H-index by the age of the publication record such that M = 1 (e.g. H = 20 after 20 years of research) is the threshold for a ‘successful’ scientist, while M = 2 is the threshold for ‘outstanding scientists’. By this measure, virtually all of the earth science projects fall below the threshold for a ‘successful’ scientist while of the SSCS projects fall above this threshold.

### Other indicators

A number of variants on the idea of the Top-1000 papers in an entire research domain have been proposed to indicate impact. One method counts the number of papers with more than 257 citations in five years yielding a one-in-1000 rate as a suggested baseline for the most successful papers. A similar analysis on space science citizen science papers and found an equivalent rate of 26 papers per 1000. Taken separately, the Top-3 papers had an average rate of 15 per 1000, while the Lower-45 had a rate of 10 per 1000. These rates are ten-fold higher than found by other investigators for non-CS research impact.

Assessing a variety of measures for productivity and impact for the earth science citizen science peer-reviewed research papers, we can tentatively conclude that the majority of the citizen science research tends to fall below the typical impact factors for natural science research, with the majority of the earth science research falling below the level of ‘successful’ research as indicated by the M-index. Nevertheless, when gauged by the presence of this research among the Top-1000 papers being cited, citizen science papers in both earth and space science areas have about 10-times the number of papers making it to this level. Some of the reasons for the lower performance may have to do with the down-turn in published papers after 2010 for the majority of the earth science projects, which itself is related to the fact that 80% of these projects only became operational after 2000 compared to the more successful ‘Top-3’ projects, whose impacts resemble the general population of non-citizen science research.

## Supporting information

S1 Data This file contains all of the data used in generating the figures, tables and conclusions for this paper. The contents are presented in seven spreadsheets identified as ‘Projects’, ‘Bibliography’, ‘Journals’, ‘Citations’, ‘eBird’, ‘GLOBE’, ‘Christmas’. The Projects tab contains a complete listing of the surveyed projects along with tabulated information about the estimated number of participants and the break down of the kinds of published documents. Bibliography lists the inventoried research papers for each project. Journals is a listing of the peer-reviewed journals in which the research articles appear. Citations is the cumulative number of citations through the end of 2017 for each bibliographic reference. The eBird, GLOBE and Christmas tabs are the separate bibliographies for the eBird, GLOBE and Christmas Bird Count projects. The tabs for Tables 1, 2 and 3 provide the data for these three tables. Fig 2 &Tables 4 and 5 provides the information for Fig 2 and Tables 4 and 5. The tabs for Figs 3, 4, 5 and 6 provide the information and tabulations for these tables. The final spreadsheet tab ‘PubDist’ provides a graph of how the full ensemble of bibliographies were distributed among specific types of documents.
(XLSX)
